# Meditation and Irritable Bowel Syndrome, a Systematic Review and Meta-Analysis

**DOI:** 10.3390/jcm11216516

**Published:** 2022-11-02

**Authors:** Cristian-Ioan Baboș, Daniel-Corneliu Leucuța, Dan Lucian Dumitrașcu

**Affiliations:** 1 Regional Institute of Gastroenterology and Hepatology, Iuliu Hațieganu University of Medicine and Pharmacy, 400158 Cluj-Napoca, Romania; 2 Medical Informatics and Biostatistics Department, Iuliu Hațieganu University of Medicine and Pharmacy, 400349 Cluj-Napoca, Romania; 3 2nd Medical Department, Faculty of Medicine, Iuliu Haţieganu University of Medicine and Pharmacy, 400000 Cluj-Napoca, Romania

**Keywords:** irritable bowel syndrome, meditation, mindfulness, self-compassion, mental healing

## Abstract

Mind-body interventions have shown efficacy in many conditions that have psychosomatic mechanisms, as well as for other pathologies. The aim of this study was to assess the effectiveness of meditation/mindfulness at improving the symptoms severity, quality of life and other associated mood and mental conditions, measured in patients with irritable bowel syndrome (IBS). A systematic review of randomized controlled trials in adult participants with IBS was conducted. Eight databases were searched for articles. We performed a meta-analysis evaluating the effects of meditation-based therapy on symptomatology, quality of life, anxiety and depression. Out of 604 articles screened, six were selected for quantitative review. The standardized mean difference (SMD) of the mindfulness group and the control group was of −36.95 (95% CI −74.61–0.7), *p* = 0.054 regarding the IBS symptom score; of 12.58 (95% CI 4.42–20.74), *p* = 0.003 regarding the IBS quality of life; SMD = 2.8 (95% CI 1.01–4.6), *p* = 0.002 for spiritual scale; and of 15.49 (95% CI −28.43–−2.55), *p* = 0.019 regarding the pain score in IBS. Our study found that the quality of life and the spiritual scale scores (i.e., mindful awareness) were statistically significantly higher in the mindfulness group, while the pain score was statistically significantly lower in the mindfulness group.

## 1. Introduction

Irritable bowel syndrome (IBS) is the condition with the highest prevalence among gastrointestinal functional diseases (between 7–10% of the general population, globally) [1]. It is estimated that about 40% of gastroenterology consultations are due to functional disorders of the digestive tract [2].

Treatment guidelines for IBS continue to be based mainly on symptom control, often unsatisfactory. Studies on this topic estimate the percentage of patients satisfied with the standard treatment received is less than 50%, many of whom resort to alternative medicine solutions [3].

Meditation is an ancient technique and tool used for mind relaxation and concentration. It is realized through a state of relaxed attention. Choosing to be aware of the mind involves mindfulness, which turns the mind inward. Mindfulness is a form of meditation whose roots can be found in secular Buddhist meditation and which has already found a well-deserved place in many areas of Western life. Mindfulness means maintaining moment-to-moment awareness of thoughts, feelings, body sensations and surroundings. Mindfulness also involves acceptance. Acceptance means paying conscious attention to thoughts and emotions without any judgementalism [4].

Meditation-based therapies, in various techniques, have been shown to be significantly effective in multiple conditions: chronic pain [5], affective disorders [6], somatization disorders, chronic inflammation in general [7].

Studies were conducted referring to the curative effect of meditation/mindfulness on the evolution of IBS. 

The aim of this systematic review was to assess the effectiveness of meditation/mindfulness at improving the symptoms severity, quality of life and other associated mood and mental conditions, measured in patients with IBS. 

## 2. Materials and Methods

### 2.1. Eligibility Criteria

We included randomized controlled studies on (P) patients with irritable bowel syndrome subjected to (I) a mindfulness intervention or (C) without mindfulness intervention to assess (O), as primary outcomes symptom severity and quality of life, as well as secondary outcomes such as stress, anxiety, spiritual scale, pain and visceral sensitivity index. Reviews, observational studies, editorials, letters to the editor and conference abstracts were excluded. 

### 2.2. Information Sources

To identify the papers of interest we accessed the following eight databases: PubMed, EMBASE, Cochrane Database, Scopus, Web of Science, Cinahl, PsychInfo, Lilacs. Reference lists of selected articles and reviews were screened for identification of other articles on the topic. 

### 2.3. Search Strategy

The search strategy included the terms: irritable bowel syndrome, meditation, mindfulness, mindfulness-based therapy (MBT), MBCT (Mindfulness Cognitive Based Therapy), MBSR (Mindfulness Based Stress Reduction Therapy), mind-body therapies, mental healing, faith healing, randomized controlled trial, along with MeSH terms, synonyms, singular and plural forms, abbreviations, as well as the Cochrane recommended search strategy for randomized controlled trials [8]. The search was performed from inception to 3 March 2022. No language restrictions were used in the search strategies, nor in article selection. The complete search strategy for each database is presented in the Supplementary Table S1. 

### 2.4. Selection Process

First, an automated elimination of duplicate studies was performed in Endnote Online, produced by Clarivate (Philadelphia, PA, USA) [9]. The remaining references were handled with Zotero, produced by Corporation for Digital Scholarship (Vienna, VA, USA) [10]. Then, two authors (CIB and DCL) manually screened the title and abstracts and excluded articles that did not meet the selection criteria, as well as duplicate studies. Disagreements were solved by discussion. Next, the same authors manually selected articles among the retrieved full text versions of the remaining articles, excluding irrelevant articles, articles of a different intended type, or duplicate studies. Disagreements were solved by discussion. 

### 2.5. Data Collection Process and Data Items

From each selected paper data was manually extracted by one author (CIB) in a Microsoft (Redmond, WA, USA) Office 365 Excel file, concerning the characteristics of the study, country, region, trial design, exposure duration, study population, age, gender, intervention, control intervention, outcomes, as well as the endpoints, symptoms severity and quality of life, including secondary outcomes such as stress, anxiety, spiritual scale, pain and visceral sensitivity index. Next, another author (DCL) rechecked the extracted data with the content of the full text paper. 

### 2.6. Study Risk of Bias Assessment

All the selected studies were assessed for presence of bias using the Cochrane Risk of bias tool 2 [11], by two authors (CIB and DCL). Disagreements were solved by discussion.

### 2.7. Effect Measures

We identified six outcome measures of interest that we assessed in our analyses: irritable bowel syndrome Severity Symptom Score (IBS-SSS), irritable bowel syndrome quality of life (IBS-QOL), irritable bowel syndrome, perceived stress (IBS-PS), irritable bowel syndrome, Spiritual Scale (FACIT-sp), irritable bowel syndrome, anxiety assessment, irritable bowel syndrome, Pain and Visceral Sensitivity. For each outcome, the mean and standard deviation were extracted and converted to standard errors. In cases where these data were missing, data were computed from confidence intervals using formulas from the Cochrane Handbook [12]. The values of interest were represented by differences of the changes, or differences of final measurements. The measurements were extracted for the end of study, as well as for the follow-up. The effect measure of interest was the standardized mean difference along with its standard error. 

### 2.8. Synthesis Methods

The mean differences and standard errors were subjected to meta-analyses using the meta package [13]. As a result of the clinical heterogeneity between the trials, the standardized mean difference and 95% confidence interval (CI) were calculated for each variable using the random effects model. The results were presented as forest plots. The chi-squared based Q-test and *I*^2^ were used to evaluate the statistical heterogeneity between the studies. The assumption of statistical significance was made if the *p*-value was less than 0.05. A leave-one-out sensitivity analysis was performed to assess the robustness of findings. The R environment for statistical computation and graphics, version 4.1.2 [14], from the R Foundation for Statistical Computing, Vienna, Austria, was used for all analyses.

### 2.9. Reporting Bias Assessment

The publication bias could not be assessed since the number of identified studies was low.

## 3. Results

**Figure 1:**
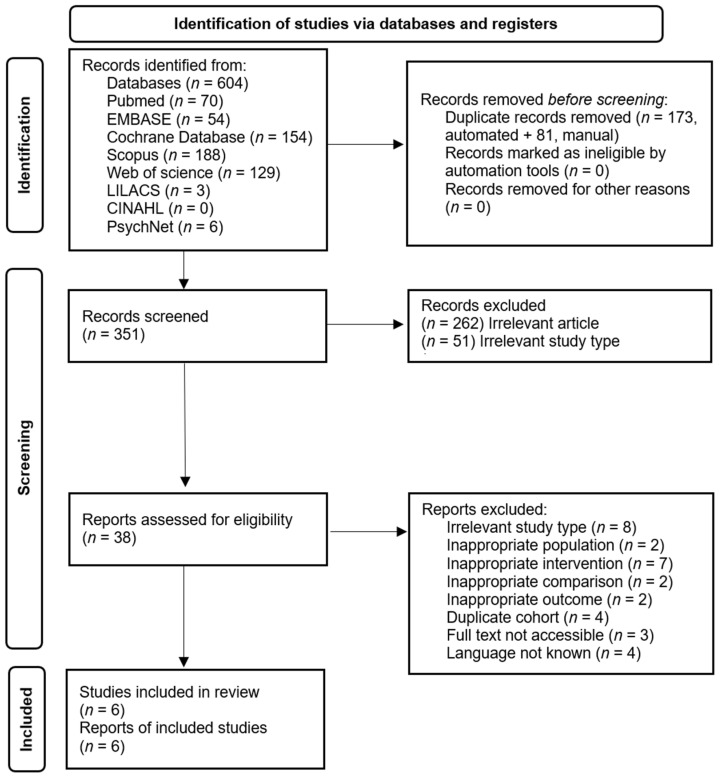
PRISMA flowchart.

A total of 604 results were retrieved from the eight searched databases. The identification and selection process is presented in Figure 1. After duplicates removal 351 records were screened and 38 remained for full-text selection assessment. Finally, six articles were included in the review and were meta-analyzed. One of the studies, from Garland et al. [15], used the same cohort of patients as Gaylord et al. [16], thus we kept only one study in our presentation, but the data was extracted from both. 

### 3.1. Study Characteristics

The study characteristics are presented in detail in Supplementary Tables S2 and S3. Two studies were conducted in North America (one in the United States of America [15] and the other in Canada [17]), and four studies were conducted in Iran [18,19,20,21]. All the studies used a parallel design and had an intervention length of eight weeks. The diagnosis of IBS for patients to be included in the studies was assessed using Rome criteria, but with different versions: one study used Rome II criteria [15], three studies used Rome III criteria [17,18,19] and two studies used Rome IV criteria [12,13]. The mean age of the patients in the studies was heterogenous; for those conducted in North America it was above 40 years and predominantly composed of females [17,20], while for the others it was around 30 years and with a similar distribution based on sex. Concerning the intervention program, five studies used Mindfulness-Based Stress Reduction (MBSR) [22], while one study used Mindfulness Based Cognitive Therapy (MBCT) [23]. All studies used group therapy. The sessions were set up as weekly sessions with a two hours time period for four studies, 2.5 h for one study [21] and 1.5 h for two studies [17,20]. Two studies additionally used a workshop retreat week of three- or four-hours length [15,17]. The training was provided by a research team member with experience with the mindfulness practice in four studies, while two studies did not mention who provided the mindfulness intervention [18,19]. Nevertheless, the years of experience varied between the studies, from two to ten years. Several studies encouraged the patients to practice at home [15,17,21]. The control group received no intervention, besides medical treatment as usual, except one study that consisted of an active support group (with discussions on IBS reported topics and subjects’ experiences and homework consisting of psychoeducational readings). Only one study used a waitlist design [17]. The inclusion criteria, beside the IBS presence, were that having a high school diploma, in case of one study [20], or age limitations, for the others. The exclusion criteria were comprehensively presented in some articles, while others did not mention them (Supplementary Table S3).

### 3.2. Treatment Outcomes

#### 3.2.1. Irritable Bowel Syndrome Severity Symptom Score 

**Figure 2:**
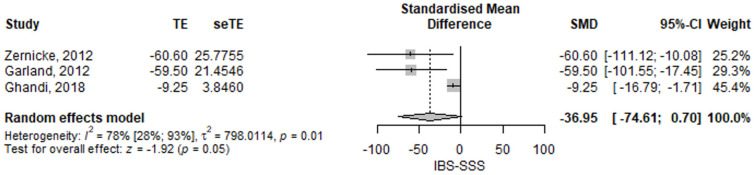
Irritable bowel syndrome symptom score—standardized mean difference comparison between mindfulness intervention and control. IBS-SSS, irritable bowel syndrome symptom score; SMD, standardized mean difference; TE, treatment effect as SMD; seTE, standard error of the treatment effect; CI, confidence interval [15,17,20].

The IBS-SSS was close to statistically significantly lower in the mindfulness group compared to the control group; the SMD of the IBS symptom score between the mindfulness group and the control group was −36.95 (95% CI −74.61–0.7), *p* = 0.054 (Figure 2). The results had high heterogeneity, *I*^2^ = 78%, *p* = 0.01. All the studies individually were statistically significant. Zernicke [17] and Garland [15] had very similar results, but Ghandi [20] had smaller differences, albeit in the same direction.

#### 3.2.2. Irritable Bowel Syndrome Quality of Life

**Figure 3:**
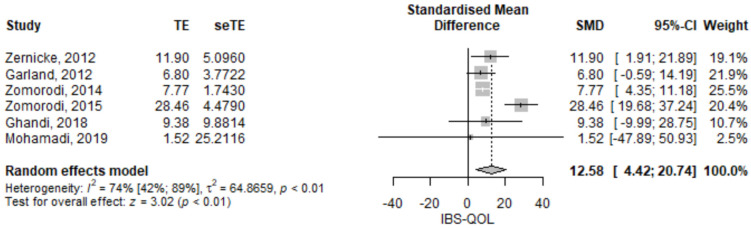
Irritable bowel syndrome quality of life—standardized mean difference comparison between mindfulness intervention and control. IBS-QOL, irritable bowel syndrome quality of life; SMD, standardized mean difference; TE, treatment effect as SMD; seTE, standard error of the treatment effect; CI, confidence interval [15,17,18,20,21].

The IBS quality of life score was statistically significant higher in the mindfulness group compared to the control group; the SMD of IBS QOL between the mindfulness group and the control group was of 12.58 (95% CI 4.42–20.74), *p* = 0.003 (Figure 3). The results had high heterogeneity, *I*^2^ = 74%, *p* < 0.01. Ghandi [20] stated that the mindfulness group had a higher QOL than the control group, but the numerical results seemed to show the contrary. Probably there was a copyediting mistake in the paper. We tried to contact the corresponding author by mail, but we did not receive any answer. Thus, the presented analysis reflects their statement. Nevertheless, we assessed the result also using the numerical results and the SMD IBS QOL pooled value was 10.17 (95% CI 0.24–20.1), *p* = 0.045, thus remaining statistically significant. Furthermore, the leave-one-out sensitivity analysis (Supplementary Figure S2), showed that, no matter which study result was excluded, the results were robust, remaining statistically significant and pointing in the same direction.

#### 3.2.3. Irritable Bowel Syndrome, Perceived Stress 

**Figure 4:**
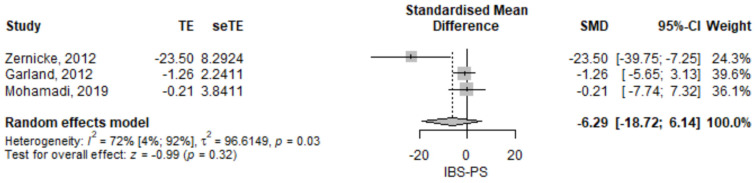
Irritable bowel syndrome—perceived stress standardized mean difference comparison between mindfulness intervention and control. IBS-PS, irritable bowel syndrome perceived stress; SMD, standardized mean difference; TE, treatment effect as SMD; seTE, standard error of the treatment effect; CI, confidence interval [15,17,21].

The perceived stress score was lower in the mindfulness group compared to the control group [SMD = −6.29 (95% CI −18.72–6.14), *p* = 0.321] (Figure 4). The results had high heterogeneity, *I*^2^ = 72%, *p* = 0.03. Zernicke found an important statistically significant difference, while Garland [15] and Mohamadi [21] reported differences close to 0. 

#### 3.2.4. Irritable Bowel Syndrome, Spiritual Scale

**Figure 5:**
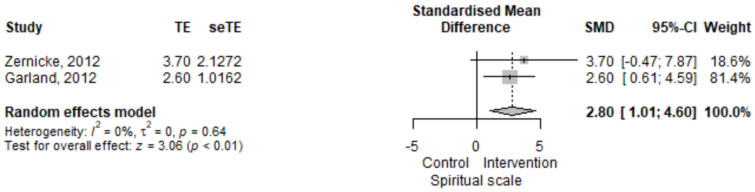
Irritable bowel syndrome the Functional Assessment of Chronic Illness Therapy—Spiritual Wellbeing Scale (FACIT-sp) standardized mean difference comparison between mindfulness intervention and control. SMD, standardized mean difference; TE, treatment effect as SMD; seTE, standard error of the treatment effect; CI, confidence interval [15,17].

The IBS observed spiritual scale score was higher in the mindfulness group compared to the control group [SMD = 2.8 (95% CI 1.01–4.6), *p* = 0.002] (Figure 5). The results had low heterogeneity, *I*^2^ = 0%, *p* = 0.64.

#### 3.2.5. Irritable Bowel Syndrome, Anxiety Assessment

**Figure 6:**
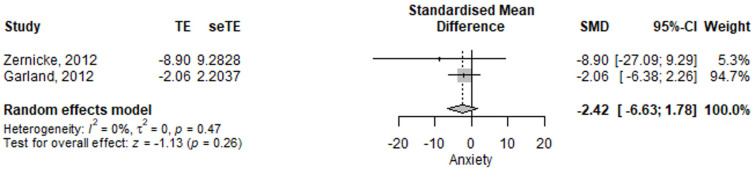
Irritable bowel syndrome anxiety scale—standardized mean difference comparison between mindfulness intervention and control. SMD, standardized mean difference; TE, treatment effect as SMD; seTE, standard error of the treatment effect; CI, confidence interval [15,17].

The IBS observed anxiety score was lower in the mindfulness group compared to the control group, albeit not reaching the significance level [−2.42 (95% CI −6.63–1.78), *p* = 0.258] (Figure 6). The results had low heterogeneity, *I*^2^ = 0%, *p* = 0.47. 

#### 3.2.6. Irritable Bowel Syndrome, Pain and Visceral Sensitivity

**Figure 7:**
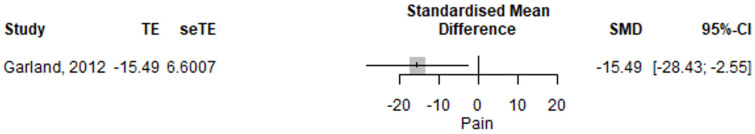
Irritable bowel syndrome pain scale—standardized mean difference comparison between mindfulness intervention and control. SMD, standardized mean difference; TE, treatment effect as SMD; seTE, standard error of the treatment effect; CI, confidence interval [15].

**Figure 8:**
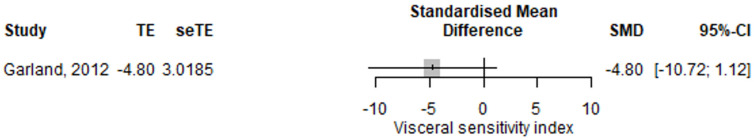
Irritable bowel syndrome visceral sensitivity index scale—standardized mean difference comparison between mindfulness intervention and control. SMD, standardized mean difference; TE, treatment effect as SMD; seTE, standard error of the treatment effect; CI, confidence interval [15].

Concerning the last outcomes, only Garland [15] reported results. The IBS observed that pain score was significantly lower in the mindfulness group compared to the control group [−15.49 (95% CI −28.43–−2.55), *p* = 0.019] (Figure 7). No differences were observed concerning the visceral sensitivity index [SMD = −4.8 (95% CI −10.72–1.12), *p* = 0.112] (Figure 8). 

### 3.3. Follow-Up Measurements

**Figure 9:**
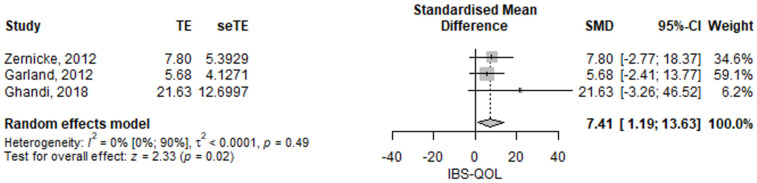
Irritable bowel syndrome quality of life at follow up,—standardized mean difference comparison between mindfulness intervention and control. IBS-QOL, irritable bowel syndrome quality of life; SMD, standardized mean difference; TE, treatment effect as SMD; seTE, standard error of the treatment effect; CI, confidence interval [15,17,20].

Only three studies reported follow-up observations after the intervention, Zernicke [17], Garland [15] and Ghandi [20]. The meta-analyses results were not statistically significant, except for IBS-QOL (Table 1). The IBS quality of life score was significantly higher in the mindfulness group compared to the control group; the SMD of IBS QOL between the mindfulness group and the control group was 7.41 (95% CI 1.19–13.63), *p* = 0.02 (Figure 9). The results had low heterogeneity, *I*^2^ = 0%, *p* = 0.49. The previously presented issue with the Ghandi study was approached similarly for follow up. Our presented analysis reflects their statement. Nevertheless, we assessed the result also using the numerical results, but the result could not be computed since the algorithm did not converge.

### 3.4. Quality Assessment

We assessed the studies using the Risk of bias tool 2 from the Cochrane Collaboration (Supplementary Figure S1). Since two studies observed the same cohort [17,18], we included them as one study in this assessment.

Concerning randomization process domain. two studies (40%) were at a high risk of bias [20,21] due to baseline differences and four had some concerns regarding bias. No study mentioned whether allocation concealment took place. Only two studies (40%) presented how randomization was undertaken [15,17].

Regarding deviations from the intended interventions two studies (40%) were at a high risk of bias [18,20], while the others had some concerns regarding bias. Although not stated in all the studies, both the participants’ carers and people delivering interventions were probably aware of the assigned intervention. No study mentioned if deviations from the intended intervention arose due to trial context. Only two studies mentioned an intention-to-treat analysis [15,17]; the rest probably used a per-protocol analysis and for one of them this approach had a potential substantial impact on the results [21].

Four studies (80%) were at high risk of bias, in respect of the missing outcome data domain, the other two having a low risk of bias. Only one study probably had data for all, or nearly all, randomized participants [21], but it is strange that this low drop out is so different compared to the other studies, which had many subjects lost to follow up (44% or 50%) [17,20]. Two studies had important percentages of subjects lost to follow up [17,20]. The other studies gave no information regarding missing data. No study mentioned any analyses to account for missing data, or sensitivity analyses, to show that the results were not biased by missing data. For all the studies, it is difficult to say if missingness in the outcome depended on its true value. The studies which had important percentages of dropouts had important differences between the intervention groups; thus it is likely that missingness in the outcome depended on its true value. 

Concerning measuring the outcome domain, four studies were at high risk of bias and one had some concerns about risk of bias. Four studies used a validated questionnaire, but one did not clarify what questionnaire it used (they stated that they used questions from Rome III). All the studies measured the outcomes with the same instruments at the same reference moments within their studies. The outcome assessor was the patient and it is likely that all the studies were not blinded to the allocated intervention. The assessment of the outcome is possibly influenced by the knowledge of the intervention received for all the studies, due to self-evaluation including judgement. One study [15] used a method to diminish this bias by having a support group to control the expectations that it provided for the same benefit as the experimental group.

Concerning the selection of the reported result domain, four studies had some concerns about bias and one was at low risk of bias [15]. This latter study analyzed the results according to a prespecified analysis plan. All the studies used only one instrument per variable of interest and only one statistical analysis method per variable of interest. 

Overall, four studies were considered at high risk of bias and only one study showed some concerns about bias [15]. 

### 3.5. Questionnaires’ Translation and Validity

Two studies used the questionnaires in the original language, American English [15,17], while the others were performed in Iran and used translations of the questionnaires. Three of the Iranian studies used validated versions of translations [18,19,20] (Supplementary Table S2). One of the Iranian studies did not clearly state whether the translation was validated, but they presented the Cronbach alpha (IBS PS α of 0.76 and IBS QOL α of 0.75).

## 4. Discussion

Our systematic search in eight databases identified six randomized controlled trials comparing mindfulness with control in subjects with IBS, in which the meta-analysis found at the end of the interventions that: the IBS symptoms severity was lower in the mindfulness group, close to statistical significance; the quality of life was statistically significant higher in the mindfulness group; the spiritual scale scores (i.e., mindful awareness) were statistically significantly higher in the mindfulness group; the pain score was statistically significantly lower in the mindfulness group; the perceived stress anxiety and visceral sensitivity index were lower in the mindfulness group, but not reaching statistical significance. Concerning the follow-up observations, the results were not statistically significant, except for IBS-QOL, which was significantly higher in the mindfulness group compared to the control group. Some of the results were characterized by an important heterogeneity. However, in general the results pointed in the same direction and in the case of IBS symptoms’ severity scores, all the individual study results were statistically significant. 

The outcomes used in the evaluated studies were measured using certain validated scales from the IBS-Severity Scoring System, which is responsive to changes in symptom severity over time. The technique uses visual analogue scales to calculate the score, which is based on five items: pain severity, pain duration, distension, bowel habit satisfaction and overall view on the quality of life [24]. IBS-Quality of Life is a 34-item instrument created at the University of Washington [25]. Scores on the measure are totaled across eight subscales on a five-point Likert scale. The labels for these subscales are dysphoria, activity disruption, negative body image, health anxiety and food avoidance, sexual influence, social reaction and relationships. The PS, Perceived Stress Scale (PSS), was created to quantify how stressful people perceive their daily circumstances to be [26]. The PSS is recommended as an outcome measure of experienced levels of stress. It is also used for exploring the role of nonspecific rated stress in the ethology of disease and behavioral disorders. The Functional Assessment of Chronic Illness Therapy—Spiritual Wellbeing Scale, a 12-item self-report questionnaire, for patients with chronic diseases, was used to assess spiritual well-being [27]. To assess anxiety two different instruments were used: BSI -18 and POMS. The Brief Symptom Inventory-18 (BSI-18) was used to gauge psychological distress (BSI-18) [28]. The BSI-18 offers distinct subscale ratings for anxiety, depression and somatization, in addition to a global symptom severity index. It assesses and quantifies the presence of the sense of calm, the sense of meaning and purpose in life. The Profile of Mood States (POMS) [29], measures six aspects of mood. The scores are related to tension-anxiety, depression-dejection, anger-hostility, vigor, exhaustion and perplexity. It has commonly been applied to medical and psychiatric populations. The POMS evaluates state (as opposed to trait) characteristics, making it suitable for repeated measurements. Pain valuation was carried out with a six-item Pain Catastrophizing Subscale of the Coping Strategy Questionnaire (CSQ) [30]. This has been demonstrated to correlate with measures of pain severity and functional impairment [31]. The Visceral Sensitivity Index (VSI), a validated 15-item scale, was used to measure hypervigilance to visceral sensations and gut-focused anxiety [32]. VSI has been demonstrated as a powerful predictor of symptom severity. 

The primary limitation is due to the risk of bias in the studies of interest. The absence of stating whether the allocation concealment was used and how randomization was performed hinders the randomization process. It is difficult to know if in reality these measures were enacted and thus the extent of the bias that is present. Using the Cochrane Risk of Bias tool, only two studies were at a high risk of bias, but if in reality the unreported methods were not protective of bias, that could modify the classification to high risk of bias for other studies too. However, several studies had similar baseline characteristics, which suggests that there bias is less likely to be present. A classical limitation in this type of studies is the difficulty of hiding the real intervention from the participants and personnel and, although a few studies stated the absence of blinding, it is implied in all the others. The missing outcome domain had a high risk of bias in many studies since it is probably difficult to allocate the time to follow the intervention. Two possible solutions might exist, one to increase compliance and the other to offer this kind of interventions to those that are more likely to follow it. The measuring of the outcome domain was at a high risk of bias in many studies, mostly since the assessment of the outcome is possible to have been influenced by the knowledge of the intervention received. In the selection of the reported result domain, the risk of bias is likely to be more limited. Another shortcoming is the small number or subjects involved in the studies; nevertheless, these kind of reviews helps to limit this by assessing more studies at once. Moreover, the results were statistically significant and were robust to sensitivity leave-one-out analyses. The questionnaires used in this review as outcomes were originally developed in American English. Two studies were caried out in English speaking countries and four in Iran. Three out of the Iranian studies used validated versions and one presented a relatively good Cronbach alpha. Using questionnaires in different languages can introduce a measurement bias and implicit comparability issues between studies, especially when the instrument is not validated (for one study it was not clear if a validated translation was used), but even for validated instruments. A selection bias was likely to have been introduced by the use of different versions of the Rome IBS diagnostic criteria (one study using Rome II, three studies using Rome III and two studies using Rome IV). The latest iteration is the Rome IV version and appears to be more precise, as evidenced by the fact that in some of the nations under examination, prevalence rates are lower than, say, under Rome III. These aspect makes the comparison of various RCTs for IBS more challenging and increases the heterogeneity of the results. Moreover, the observations were performed in different countries and cultures, that might respond differently to the interventions and induce heterogeneity in the results. 

In addition, our review has the following strengths: (1) The Cochrane Collaboration’s Risk of Bias Tool, version 2, from one of the most prestigious organizations that conducts systematic reviews and develops high-quality instruments for study validity evaluation, was used to evaluate the publications’ methodological flaws. (2) A comprehensive search strategy was used. (3) Many databases, more exactly eight (PubMed, EMBASE, Cochrane Database, Scopus, Web of Science, Cinahl, PsychInfo, Lilacs), were searched. (4) Only randomized controlled trials were included. (5) Sensitivity analyses were performed.

## 5. Conclusions

The systematic review with meta-analysis of randomized controlled trials found that the quality of life was statistically significantly higher in the mindfulness group; the spiritual scale scores (i.e., mindful awareness) were statistically significantly higher in the mindfulness group; the pain score was statistically significantly lower in the mindfulness group; the perceived stress anxiety and visceral sensitivity index were lower in the mindfulness group, but not statistically significant. The severity of the IBS symptoms was also close to being statistically significantly lower in the mindfulness group. Apart from IBS-QOL, which was statistically substantially higher in the mindfulness group as compared to the control group, the findings of the follow-up observations were not statistically significant. The analyzed trials, however, carry some methodological shortcomings, which diminish the quality of the observed evidence. Substantial improvements in methodological quality need to be implemented in future randomized controlled trials.

## Figures and Tables

**Table 1:** Meta-analyses for follow-up observations comparing mindfulness with control.

Characteristic	SMD (95% CI)	*p*-Value	Studies
IBS-SSS	−20.2 (−71.57–31.17)	0.441	[17]
IBS-QOL	7.41 (95% CI 1.19–13.63)	*p* = 0.02	[15,17,20]
IBS-PS	−2.51 (−6.7–1.69)	0.241	[15,17]
Spiritual scale	2.1 (−2.07–6.27)	0.324	[17]
Anxiety	−3.49 (−12.43–5.45)	0.445	[15,17]
Pain	−14.38 (−40.88–12.12)	0.288	[15]
Visceral sensitivity index	9.39 (−5.81–24.59)	0.226	[15]

IBS, irritable bowel syndrome; SSS, symptom severityscore; QOL, quality of life; SMD, standardized mean difference; CI, confidence interval.

## Data Availability

Data is contained within the article or Supplementary Materials.

## Supplementary Materials

The following supporting information can be downloaded at: https://www.mdpi.com/article/10.3390/jcm11216516/s1, Table S1: Search strategies; Table S2: Study characteristics; Table S3: Study inclusion and exclusion criteria; Figure S1: Quality assessment of the selected randomized controlled parallel trials using Risk of bias 2 tool from Cochrane Collaboration; Figure S2: Leave-one-out sensitivity analysis for irritable bowel syndrome quality of life standardized mean difference.
