# Supplementary material for: Meditation and Irritable Bowel Syndrome, a Systematic Review and Meta-Analysis
Source: J Clin Med. 2022 Nov 2;11(21):6516. doi: 10.3390/jcm11216516 (PMC9658118; doi:10.3390/jcm11216516)
Supplement: Supplementary file 1 [file jcm-11-06516-s001.zip › jcm-1991621-supplementary.pdf]

Supplementary file

Meditation and Irritable Bowel Syndrome—A Systematic Review and Meta-Analysis

Authors: Cristian Babos, Daniel C. Leucuta, Dan L. Dumitrascu

Supplementary Table S1. Search strategies

**Pubmed**
(("irritable bowel syndrome"[MeSH Terms] OR "irritable bowel syndrome"[All Fields] OR ("irritable"[All Fields] AND "bowel"[All Fields] AND "syndrome"[All Fields]) OR ("Colon"[All Fields] AND "Irritable"[All Fields]) OR "IBS"[TIAB])<br>AND (<br>("meditation"[MeSH Terms] OR "meditation"[All Fields] OR "meditations"[All Fields] OR "meditation's"[All Fields] OR "meditational"[All Fields] OR "meditative"[All Fields] OR "meditator"[All Fields] OR "meditators"[All Fields] OR "meditate"[All Fields] OR "meditated"[All Fields] OR "meditating"[All Fields] OR "spiritual therapy"[All Fields] OR "spiritual healing"[All Fields] OR "prayer"[All Fields] OR "tai chi"[All Fields] OR "qi gong"[All Fields])<br>OR<br>("mindfulness"[MeSH Terms] OR "mindfulness"[All Fields] OR "mindful"[All Fields] OR "Self-Compassion"[MeSH Terms] OR "MBT"[TIAB] OR "MBCT"[TIAB] OR "MBSR"[TIAB])<br>OR<br>("Mind-Body Therapies"[All Fields] OR "mind-body"[All Fields] OR "mind body"[All Fields] OR "Mental Healing"[MeSH Terms] OR "mental healing"[All Fields] OR "Faith Healing"[MeSH Terms] OR "yoga"[MeSH Terms] OR "yoga"[All Fields])))<br>AND<br>((randomized controlled trial [pt] OR controlled clinical trial [pt] OR randomized [tiab] OR placebo [tiab] OR clinical trials as topic [mesh: noexp] OR randomly [tiab] OR trial [ti]) NOT (animals [mh] NOT humans [mh]))
**EMBASE**
(('irritable colon'/exp OR 'irritable bowel syndrome' OR ('irritable' AND 'bowel' AND 'syndrome') OR ('colon' AND 'irritable') OR 'IBS':ti,ab) AND (('meditation'/exp OR 'meditation' OR 'meditations' OR 'meditational' OR 'meditative' OR 'meditator' OR 'meditators' OR 'meditate' OR 'meditated' OR 'meditating' OR 'spiritual therapy' OR 'spiritual healing' OR 'prayer' OR 'tai chi' OR 'qi gong')<br>OR ('mindfulness'/exp OR 'mindfulness' OR 'mindful' OR 'self compassion'/exp OR 'MBT':ti,ab OR 'MBCT':ti,ab OR 'MBSR':ti,ab) OR ('mind-body therapy' OR 'mind-body therapies' OR 'mind-body' OR 'mind body' OR 'mental healing' OR 'faith healing'/exp OR 'spiritual healing'/exp OR 'yoga'/exp OR 'yoga')))<br>AND (('randomized controlled trial'/exp OR 'controlled clinical study'/exp OR random$:ti,ab OR 'randomization'/exp OR 'intermethod comparison'/exp OR placebo:ti,ab OR compare:ti OR compared:ti OR comparison:ti OR ((evaluated:ab OR evaluate:ab OR evaluating:ab OR assessed:ab OR assess:ab) AND (compare:ab OR compared:ab OR comparing:ab OR comparison:ab)) OR (open:ti,ab AND adj:ti,ab AND label:ti,ab) OR ((double:ti,ab OR

single:ti,ab OR doubly:ti,ab OR singly:ti,ab) AND adj:ti,ab AND (blind:ti,ab OR blinded:ti,ab OR blindly:ti,ab)) OR 'double blind procedure'/exp OR parallel) AND group$1:ti,ab OR crossover:ti,ab OR 'cross over':ti,ab OR ((assign$:ti,ab OR match:ti,ab OR matched:ti,ab OR allocation:ti,ab) AND adj5:ti,ab AND (alternate:ti,ab OR group$1:ti,ab OR intervention$1:ti,ab OR patient$1:ti,ab OR subject$1:ti,ab OR participant$1:ti,ab)) OR assigned:ti,ab OR allocated:ti,ab OR (controlled:ti,ab AND adj7:ti,ab AND (study:ti,ab OR design:ti,ab OR trial:ti,ab)) OR volunteer:ti,ab OR volunteers:ti,ab OR 'human experiment'/exp OR trial:ti) NOT ((((((random$:ti,ab AND adj:ti,ab AND sampl$:ti,ab AND adj7:ti,ab AND ('cross section$':ti,ab OR questionnaire$1:ti,ab OR survey$:ti,ab OR database$1:ti,ab) NOT ('comparative study'/exp OR 'controlled study'/exp OR 'randomi?ed controlled':ti,ab OR 'randomly assigned':ti,ab) OR 'cross-sectional study'/exp) NOT ('randomized controlled trial'/exp OR 'controlled clinical study'/exp OR 'controlled study'/exp OR 'randomi?ed controlled':ti,ab OR 'control group$1':ti,ab) OR (case:ti,ab AND adj:ti,ab AND control$:ti,ab AND random$:ti,ab NOT 'randomi?ed controlled':ti,ab) OR ('systematic review':ti NOT (trial:ti OR study:ti)) OR (nonrandom$:ti,ab NOT random$:ti,ab) OR 'random field$':ti,ab OR ('random cluster':ti,ab AND adj3:ti,ab AND sampl$:ti,ab) OR (review:ab AND review:pt)) NOT trial:ti OR 'we searched':ab) AND (review:ti OR review:pt) OR 'update review':ab OR (databases:ab AND adj4:ab AND searched:ab) OR rat:ti OR rats:ti OR mouse:ti OR mice:ti OR swine:ti OR porcine:ti OR murine:ti OR sheep:ti OR lambs:ti OR pigs:ti OR piglets:ti OR rabbit:ti OR rabbits:ti OR cat:ti OR cats:ti OR dog:ti OR dogs:ti OR cattle:ti OR bovine:ti OR monkey:ti OR monkeys:ti OR trout:ti OR marmoset$1:ti) AND 'animal experiment'/exp OR 'animal experiment'/exp) NOT ('human experiment'/exp OR 'human'/exp))
**Cochrane Database**
("irritable bowel syndrome" OR ("irritable" AND "bowel" AND "syndrome") OR ("Colon" AND "Irritable") OR "IBS")<br>AND (("meditation" OR "meditations" OR "meditation's" OR "meditational" OR "meditative" OR "meditator" OR "meditators" OR "meditate" OR "meditated" OR "meditating" OR "spiritual therapy" OR "spiritual healing" OR "prayer" OR "tai chi" OR "qi gong")<br>OR ("mindfulness" OR "mindful" OR "Self-Compassion" OR "MBT" OR "MBCT" OR "MBSR")<br>OR ("Mind-Body Therapies" OR "mind-body" OR "mind body" OR "mental healing" OR "faith healing" OR "yoga"))
**Scopus**
TITLE-ABS-KEY("irritable bowel syndrome" OR ("irritable" AND "bowel" AND "syndrome") OR ("Colon" AND "Irritable") OR "IBS") AND TITLE-ABS-KEY(("meditation" OR "meditations" OR "meditation's" OR "meditational" OR "meditative" OR "meditator" OR "meditators" OR "meditate" OR "meditated" OR "meditating" OR "spiritual therapy" OR "spiritual healing" OR "prayer" OR "tai chi" OR "qi gong") OR ("mindfulness" OR "mindful" OR "Self-Compassion" OR "MBT" OR "MBCT" OR "MBSR") OR ("Mind-Body Therapies" OR "mind-body" OR "mind body" OR "mental healing" OR "faith healing" OR "yoga")) AND<br>("randomized controlled trial" OR ("randomized" AND "controlled" AND "trial") OR TITLE-ABS-KEY(randomized) OR TITLE-ABS-KEY(placebo) OR ("clinical" AND ("trial" OR "trials")) OR TITLE-ABS-KEY(randomly) OR TITLE(trial)) AND NOT (TITLE-ABS-KEY(rat) OR TITLE-ABS-KEY(rats) OR TITLE-ABS-KEY(mouse) OR TITLE-ABS-KEY(mice) OR TITLE-ABS-KEY(swine) OR TITLE-ABS-KEY(porcine) OR TITLE-ABS-

KEY(murine) OR TITLE-ABS-KEY(sheep) OR TITLE-ABS-KEY(lambs) OR TITLE-ABS-KEY(pigs) OR TITLE-ABS-KEY(piglets) OR TITLE-ABS-KEY(rabbit) OR TITLE-ABS-KEY(rabbits) OR TITLE-ABS-KEY(cat) OR TITLE-ABS-KEY(cats) OR TITLE-ABS-KEY(dog) OR TITLE-ABS-KEY(dogs) OR TITLE-ABS-KEY(cattle) OR TITLE-ABS-KEY(bovine) OR TITLE-ABS-KEY(monkey) OR TITLE-ABS-KEY(monkeys) OR TITLE-ABS-KEY(trout)) AND (LIMIT-TO (DOCTYPE , "ar") OR LIMIT-TO (DOCTYPE , "re") OR LIMIT-TO (DOCTYPE , "le")) AND (LIMIT-TO (SUBJAREA , "MEDI")) AND (LIMIT-TO (SRCTYPE , "j")) AND (LIMIT-TO (EXACTKEYWORD , "Human") OR LIMIT-TO (EXACTKEYWORD , "Humans"))
**Web of Science**
TS=(("irritable bowel syndrome" OR ("irritable" AND "bowel" AND "syndrome") OR ("Colon" AND "Irritable") OR "IBS") AND (("meditation" OR "meditations" OR "meditation's" OR "meditational" OR "meditative" OR "meditator" OR "meditators" OR "meditate" OR "meditated" OR "meditating" OR "spiritual therapy" OR "spiritual healing" OR "prayer" OR "tai chi" OR "qi gong") OR ("mindfulness" OR "mindful" OR "Self-Compassion" OR "MBT" OR "MBCT" OR "MBSR") OR ("Mind-Body Therapies" OR "mind-body" OR "mind body" OR "mental healing" OR "faith healing" OR "yoga"))) Filter = Article
**PsycheNet**
("irritable bowel syndrome" OR ("irritable" AND "bowel" AND "syndrome") OR ("Colon" AND "Irritable") OR "IBS")<br>AND (("meditation" OR "meditations" OR "meditation's" OR "meditational" OR "meditative" OR "meditator" OR "meditators" OR "meditate" OR "meditated" OR "meditating" OR "spiritual therapy" OR "spiritual healing" OR "prayer" OR "tai chi" OR "qi gong")<br>OR ("mindfulness" OR "mindful" OR "Self-Compassion" OR "MBT" OR "MBCT" OR "MBSR")<br>OR ("Mind-Body Therapies" OR "mind-body" OR "mind body" OR "mental healing" OR "faith healing" OR "yoga"))
**LILACS**
tw:(("irritable bowel syndrome" OR ("irritable" AND "bowel" AND "syndrome") OR ("Colon" AND "Irritable") OR "IBS")<br>AND (("meditation" OR "meditations" OR "meditation's" OR "meditational" OR "meditative" OR "meditator" OR "meditators" OR "meditate" OR "meditated" OR "meditating" OR "spiritual therapy" OR "spiritual healing" OR "prayer" OR "tai chi" OR "qi gong")<br>OR ("mindfulness" OR "mindful" OR "Self-Compassion" OR "MBT" OR "MBCT" OR "MBSR") OR ("Mind-Body Therapies" OR "mind-body" OR "mind body" OR "mental healing" OR "faith healing" OR "yoga")))
**CINAHL**
("irritable bowel syndrome" OR ("irritable" AND "bowel" AND "syndrome") OR ("Colon" AND "Irritable") OR "IBS")<br>AND (("meditation" OR "meditations" OR "meditation's" OR "meditational" OR "meditative" OR "meditator" OR "meditators" OR "meditate" OR "meditated" OR "meditating" OR "spiritual therapy" OR "spiritual healing" OR "prayer" OR "tai chi" OR "qi gong")<br>OR ("mindfulness" OR "mindful" OR "Self-Compassion" OR "MBT" OR "MBCT" OR "MBSR")<br>OR ("Mind-Body Therapies" OR "mind-body" OR "mind body" OR "mental healing" OR "faith healing" OR "yoga"))

Supplementary Table S2 Study characteristics

Study name	Country	Region	Trial design	Exposure duration	IBS assessment	Age (years), mean (SD)/median (IQR) [range] Intervention/Control	Female (%) Intervention/Control	Intervention	Control intervention	Outcome parameters and questionnaire validation
Garland, 2012 [15]	USA	North America	parallel	8 weeks	IBS Rome II	44.72 (12.55) vs. 40.89 (14.68)	100%	2h sessions weekly + one 4h retreat, based on the MBSR by Jon Kabat-Zinn at the University of Massachusetts, group therapy by a professional health coach with over 10 years of MBSR teaching. Homework assignments – daily mindful practice and psychoeducational readings	support group, weekly sessions focused on IBS-related topics and involved open group discussions about subjects' experiences on the topic. Homework assignments - psychoeducational readings	IBS SSS, IBS QOL 34, FFMQ, CSQ, VSI, BSI 18 (original versions)
Ghandi, 2018 [20]	Iran	Asia	parallel	8 weeks	IBS Rome IV	[18 to 45]	54.2%	1.5 h session of MBSR group therapy, administered by a psychotherapist	only medical therapy	IBS-SSS (persian validated), IBS-QOL-34 (persian validated)
Mohamadi, 2019 [21]	Iran	Asia	parallel	8 weeks	IBS Rome IV	28.6(3.2)/29.85(4.51)	55%/50%	2.5h weekly session, MBCT group therapy by a psychologist with over 3 years of experience in mindfulness meditation. Homework 45 min to 1 h at home by using audio CDs instructions and perform other exercises including monitoring positive or negative feelings and emotions.	no intervention	IBS PS (#, * α=0,76), IBS QOL-34 (#, * α=0,75)
Zernicke, 2013 [17]	Canada	North America	parallel	8 weeks	IBS Rome III	45 (SD=12.4)/ 44 (SD=12.6)	40 (90.3 %)/ 41 (87.2 %)	MBSR, 1.5 h session weekly group therapy by a registered nurse, a certified yoga instructor and professionally trained at university for MBSR, and teaching MBSR for two years + 3h workshop retreat week 6-7+ practice at home	waitlist, no intervention	IBS SSS, IBS QOL, C SOSI, FACIT sp (original versions)

Zomorodi, 2014 [18]	Iran	Asia	parallel	8 weeks	IBS Rome III	34.25±4.16 / 33.42±5.3	6 (50)/5(48)	MBSR, 8 sessions of group therapy, 2 hours once a week.	no intervention	IBS Q, based on Rome III (perisan validated)
Zomorodi2015 [19]	Iran	Asia	parallel	8 weeks	IBS Rome III	34/25±4/16/33/42±5/3	6 (50)/5(48)	MBSR, 8 sessions of group therapy, 2 hours once a week.	no intervention	IBS QOL-34 (perisan validated)

IBS, irritable bowel syndrome; MBSR, Mindfulness-Based Stress Reduction [7]; MBCT, Mindfulness Based Congitive Therapy [8]; SD, standard deviation; IQR, interquartile range; IBS SSS, irritable bowel syndrome Severity Symptom Score; IBS QOL, irritable bowel syndrome quality of life; FFMQ, Five Facets Mindfulness Questionnaire; CSQ, Coping Strategy Questionnaire; VSI, Visceral Sensitivity Index; BSI 18, Brief Symptom Inventory 18; IBS PS, irritable bowel syndrome, perceived stress; C SOSI, Calgary Symptoms of Stress Inventory; FACIT sp, Functional Assessment of Chronic Illness Therapy Spiritual Wellbeing Scale, irritable bowel syndrome, anxiety assessment, irritable bowel syndrome, Pain and Visceral Sensitivity; #, authors didn't specified if they used a persian validated translated version of the questionnaire; *, the Cronbach alpha calculated on the study patients.

Supplementary Table S3 Study inclusion and exclusion criteria

Study name	Inclusion criteria	Exclusion criteria
Garland, 2012 [15]	IBS, women aged 18–75 years	psychotic disorder or a history of psychiatric hospitalization in the past two years; a history of inflammatory bowel disease, celiac disease, gastrointestinal malignancy, liver or pancreatic disease, or abdominal trauma
Ghandi, 2018 [20]	IBS, age 18 to 45; having a high school diploma	psychosis, neurological disorder, and substance abuse
Mohamadi, 2019 [21]	IBS	sign of organic gastrointestinal, disease, previous gastrointestinal surgery, sign of pulmonary, renal, endocrine, cardiac, neurologic, or gynaecological pathology that probably interfered with IBS diagnosis, and diagnosis with a severe psychiatric or comorbid chronic pain according to their self-report; receiving DBT, MBCT and PPT or NFB before entering the study, taking daily medications for organic gastrointestinal symptoms or medications that act on the serotonergic, catecholaminergic, or cortisol systems and absence in the sessions
Zernicke, 2013 [17]	IBS, age 18 years or older; English-speaking	a concurrent self-reported diagnosis of a DSM-IV axis I mood, anxiety, or psychotic disorder; current use of antipsychotics; past participation in an MBSR group
Zomorodi, 2014 [18]	IBS	-
Zomorodi2015 [19]	IBS	-

Risk of bias assessment

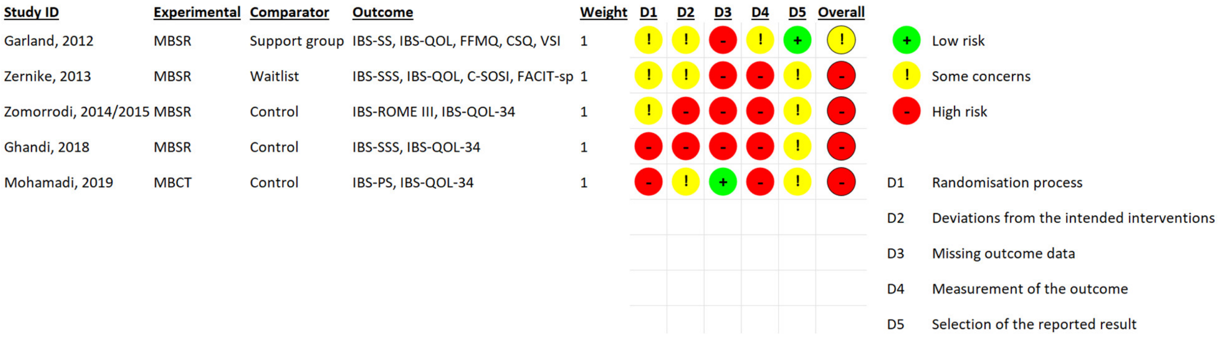

Supplementary Figure S1. Quality assessment of the selected randomized controlled parallel trials using Risk of bias 2 tool from Cochrane Collaboration [15,17–21].

Sensitivity analyses

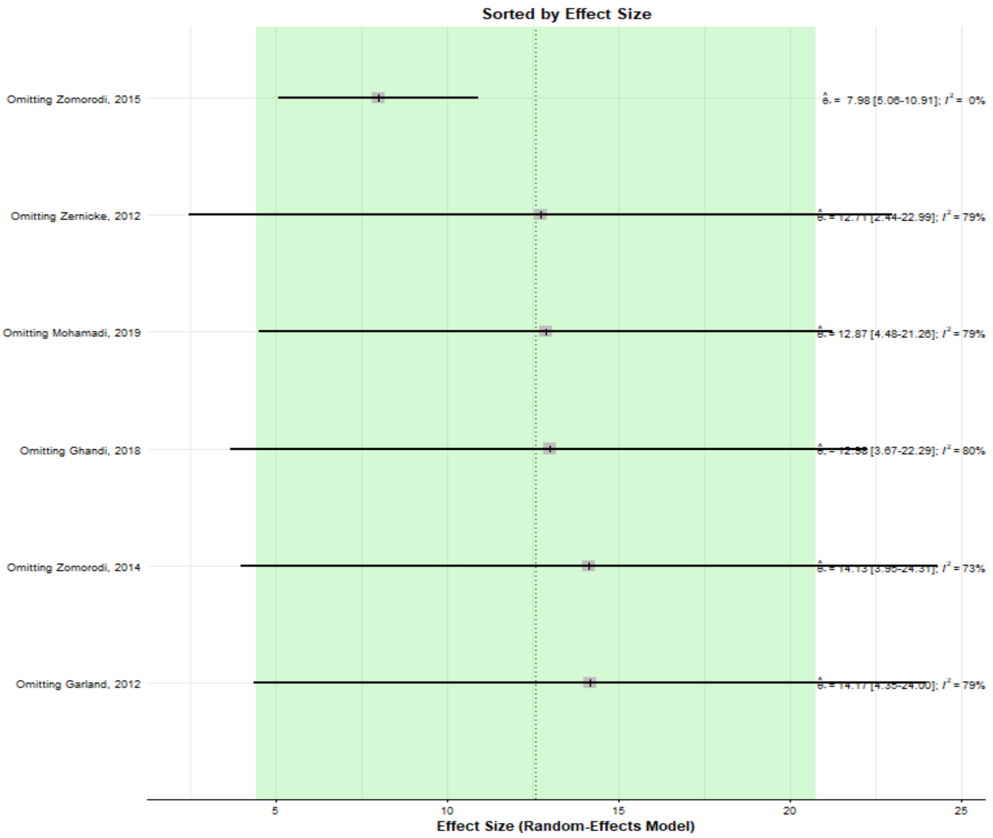

Supplementary Figure S2. Leave-one-out sensitivity analysis for irritable bowel syndrome quality of life standardized mean difference ($\hat{\Theta}$) [15,17–21].
